# Fat tissues, the brite and the dark sides

**DOI:** 10.1007/s00424-016-1884-8

**Published:** 2016-10-04

**Authors:** Yong Chen, Ruping Pan, Alexander Pfeifer

**Affiliations:** 1 Institute of Pharmacology and Toxicology, University Hospital Bonn, University of Bonn, 53127 Bonn, Germany; 2 Department of Cell and Tissue Biology, UCSF Diabetes Center, University of California, San Francisco, CA 94143-0669 USA

**Keywords:** Metabolism, Obesity, Brown adipose tissue, Brite/beige adipocytes, Energy expenditure

## Abstract

Fat tissue is well known for its capacity to store energy and its detrimental role in obesity and metaflammation. However, humans possess different types of fat that have different functions in physiology and metabolic diseases. Apart from white adipose tissue (WAT), the body’s main energy storage, there is also brown adipose tissue (BAT) that dissipates energy as a defense against cold and maintains energy balance for the whole body. BAT is present not only in newborns but also in adult humans and its mass correlates with leanness. Moreover, “brown-like” adipocytes have been detected in human WAT. These “brown-in-white” (brite) or beige cells can be induced by cold and a broad spectrum of pharmacological substances and, therefore, they are also known as “inducible brown adipocytes.” Activation of brown and/or brite adipocytes reduces metabolic diseases, at least in murine models of obesity. Thus, brown/brite adipocytes represent the “brite” side of fat and are potential targets for novel therapeutic approaches for treatment of obesity and obesity-associated diseases.

## Introduction

Obesity and overweight have been conclusively shown to increase the risk of type 2 diabetes, hypertension, hypercholesterolemia, cardiovascular disease, and certain types of cancer [27]. Obesity not only affects people in developed countries but also people in developing countries who intake large amounts of calorie-dense food. The World Health Organization (WHO) reported in 2014 that 39 % of adults worldwide were overweight and 13 % were obese. Overweight and obesity are urgent health issues: the numbers have doubled since 1990 and—according to the WHO—more people die of obesity and its consequences than of undernutrition and famine.

An important pathophysiological basis of obesity and obesity-associated disorders is the increase in adipocyte size due to the uptake/storage of excess energy in the form of lipids, which causes cellular stress and an inflammatory response that spreads throughout the whole body (metaflammation) [19].

In addition to the “bad” white fat that represents, for many people, the “dark side” of fat tissue, there is also a bright side: brown fat takes up glucose and lipids and burns energy to generate heat. Moreover, cold exposure and different pharmacological stimuli bring out “brown-like” cells in white fat depots. These cells function similar as brown adipocytes and are also termed brite or beige cells.

## Types of fat

### White fat

White adipose tissue (WAT) is the major adipose organ in adults and is the main storage site of energy in the form of triacylglycerols. Whenever fuel is required, fatty acids are released from WAT by lipolysis. This process is initiated by norepinephrine which binds to the beta-adrenergic receptors on white adipocytes leading to the generation of cAMP. The second messenger cAMP activates protein kinase A and, in turn, stimulates the hormone-sensitive lipase releasing free fatty acids from triacylglycerol in the lipid droplets. WAT composes as much as 20 % of the body weight of healthy adult humans. Although it is widely distributed throughout the whole body, separate/specific depots can be distinguished: visceral white adipose tissue (vWAT) mainly surrounds internal organs, whereas superficial or inguinal white adipose tissue (igWAT) is located beneath the skin.

Morphologically, a white adipocyte is a unilocular cell and contains a single large lipid droplet that pushes the nucleus close to the plasma membrane. Mitochondria are located mainly in the thicker portion of the cytoplasmic rim near the nucleus. Beyond simple fat storage, WAT is also a secretory and endocrine organ that secretes hormones (including leptin, adiponectin, angiotensinogen, tumor necrosis factor α (TNFα), interleukin 6 (IL-6), metallothionein, resistin, and etc.) and has an important role in metabolic homeostasis, inflammatory processes, and vascular homeostasis [46]. Although no specific markers for WAT have been identified, several genes including fatty-acid binding protein 4 (FABP4 or aP2), peroxisome proliferator-activated receptor γ (PPARγ), and CCAAT/enhancer-binding protein α (C/EBPα) have been found to have an important role and/or are highly expressed in WAT.

Development of WAT is initiated in the mesoderm during the embryonic period [12]. Murine igWAT develops between embryonic days 14 and 18, whereas vWAT develops postnatally [54]. White adipocytes have been thought to originate from precursors that lack myogenic factor 5 (Myf5) [5, 7, 48], until Guertin’s group discovered different origins of white adipocytes from different WAT depots: white adipocytes from posterior subcutaneous, mesenteric, and perigonadal visceral depots are all Myf5-negative, whereas those from anterior subcutaneous and retroperitoneal visceral depots are nearly all Myf5-positive [39].

The transcriptional regulation of WAT adipogenesis involves the activation of several transcriptional factors including PPARγ and C/EBPs [36]. PPARγ is a master regulator of all kinds/colors of adipose tissue and is indispensable for WAT development [2, 37]. C/EBPα maintains expression of PPARγ and, together with PPARγ, regulates gene transcription to promote adipocyte differentiation. Consequently, C/EBPα deficiency in mice inhibits WAT development [23].

### Brown fat

Brown adipose tissue (BAT) is a special type of adipose organ found in almost all mammals including mice, rats, rabbits, sheep, bears, and humans [44]. Pigs are one exception: they lack BAT and are completely dependent on shivering thermogenesis to keep warm [47]. Activated BAT burns lipids and glucose, contributing to energy dissipation and thus results in heat production, a process known as non-shivering thermogenesis (NST). NST is critically dependent on uncoupling protein 1 (UCP1), a brown adipocyte-specific protein. UCP1 uncouples the respiratory chain of oxidative phosphorylation within mitochondria, shifts energy from the mitochondrial electron chain away from ATP production, and releases the superfluent energy as heat. BAT has been thought to exist only in hibernating mammals and newborns, until functional BAT was discovered in adult humans in 2009 [6, 38, 50, 51]. Regarding its location, unlike WAT, its distribution is limited mainly in the supraclavicular, neck, and perirenal regions of human body [28, 45]. In comparison to lipid-loaded, mature white adipocytes, brown adipocytes are smaller and contain multilocular smaller lipid droplets and many UCP1 positive mitochondria, which vary in size and shape and are a major reason for the color of BAT. Apart from *UCP1*, several markers for brown adipocytes have been described including, peroxisome-proliferator-activated receptor γ-coactivator 1α (*PGC-1α*), cell death-inducing DNA fragmentation factor alpha-like effector A (*Cidea*), *Zic1*, *Lhx8*, *Eva1*, and *Epsti1* [11].

BAT develops earlier than WAT during embryogenesis (as early as day 9.5) [3, 22, 55]. Brown adipocytes arise from central dermomyotome during embryonic development, and they share their origin with skeletal muscle cells, dermal cells, and a subpopulation of white adipocytes [1, 22, 40, 41]. Although Myf5 was initially reported as a specific marker for precursors that give rise to brown adipocytes and muscle cells [41], recent studies in mice showed that Myf5-positive precursors also give rise to white adipocytes in anterior/dorsal depots indicating that Myf5 is rather a marker for cell position [39].

During BAT development, some positive regulators have been identified so far, including protein PR domain containing 16 (PRDM16), PPARα, bone morphogenetic protein 7 (BMP7) and Orexin. Although PRDM16 was shown to be dispensable for brown adipocyte development [4], it is required for maintaining BAT function during aging. PRDM16 forms complexes with other regulatory factors including PPARγ, PGC-1α/β, euchromatic histone-lysine *N*-methyltransferase 1 (EHMT1), C-terminal-binding proteins (CtBPs), and early B cell factor-2 (EBF2) [20, 21, 30, 34, 41, 42]. PPARα was demonstrated to bind to a PPAR-responsive element in the distal PGC-1α gene promoter, thereby, inducing expression of PGC-1α [17]. BMP7 and Orexin have been shown to promote brown adipocyte development via induction of PGC-1α, UCP1, PPARγ, and C/EBPs [49] as well as through p38 mitogen-activated protein kinase (MAPK) and bone morphogenetic protein receptor-1α (BMPR1A)-dependent Smad 1/5 signaling [43].

### Brite fat

**Fig. 1:**
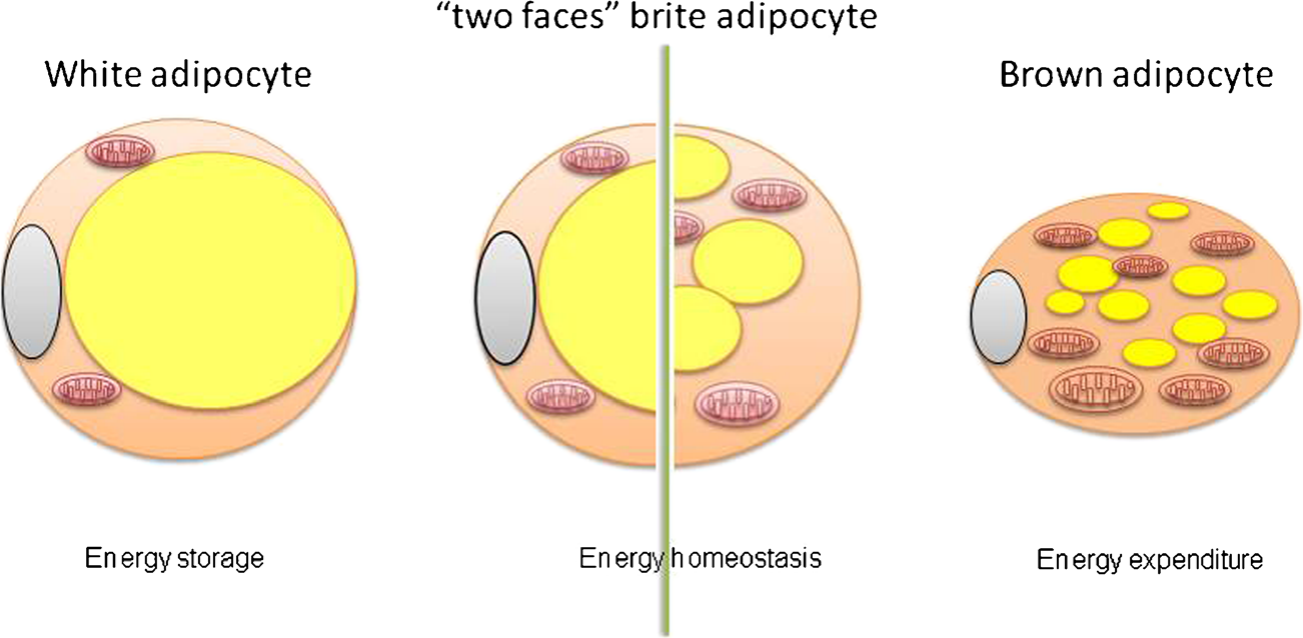
White, brite, brown adipocytes and the two faces of brite adipocytes

Brite/beige fat, which is also known as inducible brown adipose tissue, functions as an extra or reserve brown adipose tissue that can be induced by cold exposure to dissipate energy [15, 32]. Brite adipocytes are dispersed among the white adipocytes and are morphologically similar to a classical brown adipocyte (Fig. 1) containing multilocular, but variable-in-size, lipid droplets and plenty of UCP1-positive mitochondria [57, 58]. Brite cells also express brown fat-specific genes, including *UCP1*, *Cidea*, *PGC-1α*, *PRDM16*, and CCAAT/enhancer-binding protein β (*C/EBPβ*). In mice, Zic1 and Hoxc9 have been identified as the most specific markers for classical BAT and brite fat, respectively [53]. In addition, several other potential brite-selective markers including *Cd137*, *Tbx1*, *Tmem26*, *Cited1*, and *Shox2* have been suggested [11].

There is no consensus concerning the mechanism of “browning” and the embryonic origin of brite adipocytes. There is evidence that brite adipocytes arise from pre-existing white adipocytes [13, 52]. On the other hand, there is also evidence that they arise by de novo adipogenesis from precursors [54]. Moreover, it was postulated that brite cells are masked as white adipocytes and might “de-mask” upon cold exposure or pharmacological stimulation [29]. Interestingly, another study showed about 10 % of brite adipocytes in igWAT arise from smooth muscle [24]. Thus, brite adipocytes might be more heterogenous than other adipocytes.

PRDM16 and PPARα/γ play critical roles in brite cell development. Their positive regulatory effect has been shown to be related to an induction in PGC-1α expression [16, 56] and a stable interaction of PRDM16 and PPARγ, which might be promoted by a Sirtuin 1 (SIRT1)-dependent deacetylation of PPARγ [33]. In addition, the cyclic GMP (cGMP) pathway has been shown to induce brite adipocyte development as well [14, 15, 25].

## Prospect and challenges

Since white fat is often viewed as “bad” or as the “dark side” of adipose tissue, one might be inclined to overcome or ease obesity via inhibition of adipose tissue expansion [10, 26]. It sounds like a reasonable therapeutic approach, since many regulatory factors have been identified that regulate differentiation of precursor cells to mature adipocytes. However, evidence from several animal models [8, 26, 35] show that blocking adipocyte development is unhealthy. If lipids are not stored by adipose tissue, they “spill over” and are stored ectopically. Ectopic storage of excess lipids in the liver and muscle is detrimental for these tissues and will worsen the metabolic dysfunction [10]. Moreover, adipose tissue functions as a secretory organ and secretes hormones like leptin and adiponectin that play important roles in appetite regulation and cardiovascular health, respectively [9, 18]. For these reasons, it is clear that other ways to fight obesity are needed.

An alternative might be to further the “brite side” of fat by increasing the number of brown and/or of brite cells. According to the evidence of numerous animal models [4, 11, 14, 15, 25, 31–33, 53], several regulatory factors of brown and brite fat development might be used for such an approach. However, there is a lack of human studies on this subject. A major reason for this is the lack of easy accessible biomarkers for brown and brite fat in humans. It is also not known whether there might be unwanted side effects of a long-term enhancement of thermogenesis. Thus, more human studies are needed to unravel the role of human brown and brite fat in physiology and disease.

## References

[1] Atit R, Sgaier SK, Mohamed OA, Taketo MM, Dufort D, Joyner AL, Niswander L, Conlon RA (2006). Beta-catenin activation is necessary and sufficient to specify the dorsal dermal fate in the mouse. Dev Biol.

[2] Barak Y, Nelson MC, Ong ES, Jones YZ, Ruiz-Lozano P, Chien KR, Koder A, Evans RM (1999). PPAR gamma is required for placental, cardiac, and adipose tissue development. Mol Cell.

[3] Cannon B, Nedergaard J (2004). Brown adipose tissue: function and physiological significance. Physiol Rev.

[4] Cohen P, Levy JD, Zhang Y, Frontini A, Kolodin DP, Svensson KJ, Lo JC, Zeng X, Ye L, Khandekar MJ, Wu J, Gunawardana SC, Banks AS, Camporez JP, Jurczak MJ, Kajimura S, Piston DW, Mathis D, Cinti S, Shulman GI, Seale P, Spiegelman BM (2014). Ablation of PRDM16 and beige adipose causes metabolic dysfunction and a subcutaneous to visceral fat switch. Cell.

[5] Cristancho AG, Lazar MA (2011). Forming functional fat: a growing understanding of adipocyte differentiation. Nat Rev Mol Cell Biol.

[6] Cypess AM, Lehman S, Williams G, Tal I, Rodman D, Goldfine AB, Kuo FC, Palmer EL, Tseng YH, Doria A, Kolodny GM, Kahn CR (2009). Identification and importance of brown adipose tissue in adult humans. N Engl J Med.

[7] Enerback S (2009). The origins of brown adipose tissue. N Engl J Med.

[8] Fairbridge NA, Southall TM, Ayre DC, Komatsu Y, Raquet PI, Brown RJ, Randell E, Kovacs CS, Christian SL (2015). Loss of CD24 in mice leads to metabolic dysfunctions and a reduction in white adipocyte tissue. PLoS One.

[9] Friedman JM (2010). A tale of two hormones. Nat Med.

[10] Gastaldelli A (2011). Role of beta-cell dysfunction, ectopic fat accumulation and insulin resistance in the pathogenesis of type 2 diabetes mellitus. Diabetes Res Clin Pract.

[11] Harms M, Seale P (2013). Brown and beige fat: development, function and therapeutic potential. Nat Med.

[12] Hausman GJ, Campion DR (1982). Histology of the stroma in developing rat subcutaneous adipose tissue. J Anim Sci.

[13] Himms-Hagen J, Melnyk A, Zingaretti MC, Ceresi E, Barbatelli G, Cinti S (2000). Multilocular fat cells in WAT of CL-316243-treated rats derive directly from white adipocytes. Am J Physiol Cell Physiol.

[14] Hoffmann LS, Etzrodt J, Willkomm L, Sanyal A, Scheja L, Fischer AW, Stasch JP, Bloch W, Friebe A, Heeren J, Pfeifer A (2015). Stimulation of soluble guanylyl cyclase protects against obesity by recruiting brown adipose tissue. Nat Commun.

[15] Hoffmann LS, Larson CJ, Pfeifer A (2015). cGMP and brown adipose tissue. Handb Exp Pharmacol.

[16] Hondares E, Mora O, Yubero P, Rodriguez de la Concepcion M, Iglesias R, Giralt M, Villarroya F (2006). Thiazolidinediones and rexinoids induce peroxisome proliferator-activated receptor-coactivator (PGC)-1alpha gene transcription: an autoregulatory loop controls PGC-1alpha expression in adipocytes via peroxisome proliferator-activated receptor-gamma coactivation. Endocrinology.

[17] Hondares E, Rosell M, Diaz-Delfin J, Olmos Y, Monsalve M, Iglesias R, Villarroya F, Giralt M (2011). Peroxisome proliferator-activated receptor alpha (PPARalpha) induces PPARgamma coactivator 1alpha (PGC-1alpha) gene expression and contributes to thermogenic activation of brown fat: involvement of PRDM16. J Biol Chem.

[18] Hopkins TA, Ouchi N, Shibata R, Walsh K (2007). Adiponectin actions in the cardiovascular system. Cardiovasc Res.

[19] Hotamisligil GS (2006). Inflammation and metabolic disorders. Nature.

[20] Kajimura S, Seale P, Kubota K, Lunsford E, Frangioni JV, Gygi SP, Spiegelman BM (2009). Initiation of myoblast to brown fat switch by a PRDM16-C/EBP-beta transcriptional complex. Nature.

[21] Kajimura S, Seale P, Tomaru T, Erdjument-Bromage H, Cooper MP, Ruas JL, Chin S, Tempst P, Lazar MA, Spiegelman BM (2008). Regulation of the brown and white fat gene programs through a PRDM16/CtBP transcriptional complex. Genes Dev.

[22] Lepper C, Fan CM (2010). Inducible lineage tracing of Pax7-descendant cells reveals embryonic origin of adult satellite cells. Genesis.

[23] Linhart HG, Ishimura-Oka K, Demayo F, Kibe T, Repka D, Poindexter B, Bick RJ, Darlington GJ (2001). C/EBPalpha is required for differentiation of white, but not brown, adipose tissue. Proc Natl Acad Sci U S A.

[24] Long JZ, Svensson KJ, Tsai L, Zeng X, Roh HC, Kong X, Rao RR, Lou J, Lokurkar I, Baur W, Castellot JJ, Rosen ED, Spiegelman BM (2014). A smooth muscle-like origin for beige adipocytes. Cell Metab.

[25] Mitschke MM, Hoffmann LS, Gnad T, Scholz D, Kruithoff K, Mayer P, Haas B, Sassmann A, Pfeifer A, Kilic A (2013). Increased cGMP promotes healthy expansion and browning of white adipose tissue. FASEB J.

[26] Moitra J, Mason MM, Olive M, Krylov D, Gavrilova O, Marcus-Samuels B, Feigenbaum L, Lee E, Aoyama T, Eckhaus M, Reitman ML, Vinson C (1998). Life without white fat: a transgenic mouse. Genes Dev.

[27] Must A, Spadano J, Coakley EH, Field AE, Colditz G, Dietz WH (1999). The disease burden associated with overweight and obesity. JAMA.

[28] Nedergaard J, Bengtsson T, Cannon B (2007). Unexpected evidence for active brown adipose tissue in adult humans. Am J Physiol Endocrinol Metab.

[29] Nedergaard J, Cannon B (2014). The browning of white adipose tissue: some burning issues. Cell Metab.

[30] Ohno H, Shinoda K, Ohyama K, Sharp LZ, Kajimura S (2013). EHMT1 controls brown adipose cell fate and thermogenesis through the PRDM16 complex. Nature.

[31] Perino A, Beretta M, Kilic A, Ghigo A, Carnevale D, Repetto IE, Braccini L, Longo D, Liebig-Gonglach M, Zaglia T, Iacobucci R, Mongillo M, Wetzker R, Bauer M, Aime S, Vercelli A, Lembo G, Pfeifer A, and Hirsch E. Combined inhibition of PI3Kbeta and PI3Kgamma reduces fat mass by enhancing alpha-MSH-dependent sympathetic drive. Sci Signal 7: ra110, 2014. 10.1126/scisignal.2005485 25406378

[32] Pfeifer A, Hoffmann LS (2014). Brown, beige, and white: the new color code of fat and its pharmacological implications. Annu Rev Pharmacol Toxicol.

[33] Qiang L, Wang L, Kon N, Zhao W, Lee S, Zhang Y, Rosenbaum M, Zhao Y, Gu W, Farmer SR, Accili D (2012). Brown remodeling of white adipose tissue by SirT1-dependent deacetylation of Ppargamma. Cell.

[34] Rajakumari S, Wu J, Ishibashi J, Lim HW, Giang AH, Won KJ, Reed RR, Seale P (2013). EBF2 determines and maintains brown adipocyte identity. Cell Metab.

[35] Reitman ML, and Gavrilova O. A-ZIP/F-1 mice lacking white fat: a model for understanding lipoatrophic diabetes. Int J Obes Relat Metab Disord 24 Suppl 4: S11-14, 2000. 10.1038/sj.ijo.0801493 11126232

[36] Rosen ED, Macdougald OA (2006). Adipocyte differentiation from the inside out. Nat Rev Mol Cell Biol.

[37] Rosen ED, Sarraf P, Troy AE, Bradwin G, Moore K, Milstone DS, Spiegelman BM, Mortensen RM (1999). PPAR gamma is required for the differentiation of adipose tissue in vivo and in vitro. Mol Cell.

[38] Saito M, Okamatsu-Ogura Y, Matsushita M, Watanabe K, Yoneshiro T, Nio-Kobayashi J, Iwanaga T, Miyagawa M, Kameya T, Nakada K, Kawai Y, Tsujisaki M (2009). High incidence of metabolically active brown adipose tissue in healthy adult humans: effects of cold exposure and adiposity. Diabetes.

[39] Sanchez-Gurmaches J, Guertin DA (2014). Adipocytes arise from multiple lineages that are heterogeneously and dynamically distributed. Nat Commun.

[40] Sanchez-Gurmaches J, Hung CM, Sparks CA, Tang Y, Li H, Guertin DA (2012). PTEN loss in the Myf5 lineage redistributes body fat and reveals subsets of white adipocytes that arise from Myf5 precursors. Cell Metab.

[41] Seale P, Bjork B, Yang W, Kajimura S, Chin S, Kuang S, Scime A, Devarakonda S, Conroe HM, Erdjument-Bromage H, Tempst P, Rudnicki MA, Beier DR, Spiegelman BM (2008). PRDM16 controls a brown fat/skeletal muscle switch. Nature.

[42] Seale P, Kajimura S, Yang W, Chin S, Rohas LM, Uldry M, Tavernier G, Langin D, Spiegelman BM (2007). Transcriptional control of brown fat determination by PRDM16. Cell Metab.

[43] Sellayah D, Bharaj P, Sikder D (2011). Orexin is required for brown adipose tissue development, differentiation, and function. Cell Metab.

[44] Smith RE, Horwitz BA (1969). Brown fat and thermogenesis. Physiol Rev.

[45] Tanuma Y, Tamamoto M, Ito T, Yokochi C (1975). The occurrence of brown adipose tissue in perirenal fat in Japanese. Arch Histol Jpn.

[46] Trayhurn P, Beattie JH (2001). Physiological role of adipose tissue: white adipose tissue as an endocrine and secretory organ. Proc Nutr Soc.

[47] Trayhurn P, Temple NJ, Van Aerde J (1989). Evidence from immunoblotting studies on uncoupling protein that brown adipose tissue is not present in the domestic pig. Can J Physiol Pharmacol.

[48] Tseng YH, Cypess AM, Kahn CR (2010). Cellular bioenergetics as a target for obesity therapy. Nat Rev Drug Discov.

[49] Tseng YH, Kokkotou E, Schulz TJ, Huang TL, Winnay JN, Taniguchi CM, Tran TT, Suzuki R, Espinoza DO, Yamamoto Y, Ahrens MJ, Dudley AT, Norris AW, Kulkarni RN, Kahn CR (2008). New role of bone morphogenetic protein 7 in brown adipogenesis and energy expenditure. Nature.

[50] van Marken Lichtenbelt WD, Vanhommerig JW, Smulders NM, Drossaerts JM, Kemerink GJ, Bouvy ND, Schrauwen P, Teule GJ (2009). Cold-activated brown adipose tissue in healthy men. N Engl J Med.

[51] Virtanen KA, Lidell ME, Orava J, Heglind M, Westergren R, Niemi T, Taittonen M, Laine J, Savisto NJ, Enerback S, Nuutila P (2009). Functional brown adipose tissue in healthy adults. N Engl J Med.

[52] Vitali A, Murano I, Zingaretti MC, Frontini A, Ricquier D, Cinti S (2012). The adipose organ of obesity-prone C57BL/6J mice is composed of mixed white and brown adipocytes. J Lipid Res.

[53] Walden TB, Hansen IR, Timmons JA, Cannon B, Nedergaard J (2012). Recruited vs. nonrecruited molecular signatures of brown, “brite,” and white adipose tissues. Am J Physiol Endocrinol Metab.

[54] Wang QA, Tao C, Gupta RK, Scherer PE (2013). Tracking adipogenesis during white adipose tissue development, expansion and regeneration. Nat Med.

[55] Wang W, Kissig M, Rajakumari S, Huang L, Lim HW, Won KJ, Seale P (2014). Ebf2 is a selective marker of brown and beige adipogenic precursor cells. Proc Natl Acad Sci U S A.

[56] Wilson-Fritch L, Nicoloro S, Chouinard M, Lazar MA, Chui PC, Leszyk J, Straubhaar J, Czech MP, Corvera S (2004). Mitochondrial remodeling in adipose tissue associated with obesity and treatment with rosiglitazone. J Clin Invest.

[57] Wu J, Bostrom P, Sparks LM, Ye L, Choi JH, Giang AH, Khandekar M, Virtanen KA, Nuutila P, Schaart G, Huang K, Tu H, van Marken Lichtenbelt WD, Hoeks J, Enerback S, Schrauwen P, Spiegelman BM (2012). Beige adipocytes are a distinct type of thermogenic fat cell in mouse and human. Cell.

[58] Xue B, Rim JS, Hogan JC, Coulter AA, Koza RA, Kozak LP (2007). Genetic variability affects the development of brown adipocytes in white fat but not in interscapular brown fat. J Lipid Res.

